# Biocontrol of *Melolontha* spp. Grubs in Organic Strawberry Plantations by Entomopathogenic Fungi as Affected by Environmental and Metabolic Factors and the Interaction with Soil Microbial Biodiversity

**DOI:** 10.3390/insects12020127

**Published:** 2021-02-02

**Authors:** Malgorzata Tartanus, Ewa M. Furmanczyk, Loredana Canfora, Flavia Pinzari, Cezary Tkaczuk, Anna Majchrowska-Safaryan, Eligio Malusá

**Affiliations:** 1 Research Institute of Horticulture-NRI, Department of Plant Protection against Pests, 96-100 Skierniewice, Poland; malgorzata.tartanus@inhort.pl (M.T.); ewa.furmanczyk@inhort.pl (E.M.F.); 2 Centre for Agriculture and Environment, Council for Agricultural Research and Economics, 00-184 Rome, Italy; loredana.canfora@crea.gov.it (L.C.); flavia.pinzari@cnr.it (F.P.); 3 Institute for Biological Systems, Council of National Research of Italy (CNR), 00-015 Monterotondo, Italy; 4 Institute of Agriculture and Horticulture, Siedlce University of Natural Sciences and Humanities, 08-110 Siedlce, Poland; cezary.tkaczuk@uph.edu.pl (C.T.); anna.majchrowska-safaryan@uph.edu.pl (A.M.-S.)

**Keywords:** *Beauveria bassiana*, *Beauveria brongniartii*, European cockchafer, chitinolytic activity, organic farming

## Abstract

**Simple Summary:**

The study aimed at relating some metabolic characteristics of two entomopathogenic fungi (*Beauveria* *bassiana* and *B. brongniartii)*, critical for their virulence and persistence in soil, to the strain’s capacity to reduce the damage of *Melolontha* sp. grubs in two organic strawberry plantations. Combination of the two species was also tested to achieve a higher efficacy, due to their different living behaviors. This hypothesis was not confirmed in the study, probably due to the *B. bassiana* metabolic competitive advantage emerged from the phenotypic characterization. The strong in vitro metabolic activity of the *B. bassiana* strain could be also associated to the higher abundance of this species in the inoculated soils in comparison to *B. brongniartii* strain. Considering the impact on soil biodiversity, the inoculation with both strains or the co-inoculum did not affect the natural fungal and bacteria communities in the soil, according to terminal restriction fragment length polymorphism (TRFLPs) analysis and qPCR data. The study provides a complex view of the effects of bioinocula to plant protection and soil biodiversity, taking into consideration the mechanisms of fungal virulence and the effect of environmental conditions on them.

**Abstract:**

The efficacy of two strains of two *Beauveria* species (*B. bassiana* and *B. brongniartii)*, individually or as co-inoculants, to control *Melolontha* sp. grubs was assessed in two organic strawberry plantations in relation to the environmental conditions, their abundance after soil inoculation, and their in vitro chitinolytic activity, thereby also verifying their impact on soil microbial communities. A reduction of the grubs’ damage to strawberry plants was observed when compared to the untreated control in one plantation, irrespective of the strain used and whether they were applied as single or as co-inoculum. The metabolic pattern expressed by the two fungi in vitro was different: *B. bassiana* showed a higher metabolic versatility in the use of different carbon sources than *B. brongniartii*, whose profile was partly overlapped in the co-inoculum. Similar differences in the chitinolytic activity of each of the fungi and the co-inoculum were also pointed out. A higher abundance of *B. bassiana* in the soils receiving this species in comparison to those receiving *B. brongniartii*, together with its in vitro metabolic activity, could account for the observed diverse efficacy of pest damage control of the two species. However, environmental and climatic factors also affected the overall efficacy of the two bioinocula. According to the monitoring of the two species in soil, *B. bassiana* could be considered as a common native species in the studied locations in contrast to *B. brongniartii*, which seemed to be a non-endemic species. Nevertheless, the inoculation with both species or the co-inoculum did not consistently affect the soil microbial (fungi and bacteria) biodiversity, as expressed by the operational taxonomic unit (OTU) number and Shannon–Wiener diversity index based on terminal restriction fragment length polymorphism (TRFLP) data. A small transient increase of the share of the inoculated species to the total fungal community was noted by the analysis of genes copy numbers only for *B. brongniartii* at the end of the third growing season.

## 1. Introduction

The grubs of the European cockchafer (*Melolontha melolontha* L.) and the forest cockchafer (*Melolontha hippocastani* Fabr.) are causing severe damage and economic losses in agriculture, horticulture, viticulture, and forestry in many European countries [1,2]. The damage derives from the feeding behavior of the grubs on the plant root system, particularly from the last instar larvae [3]. The lack of chemical formulations to control both larval and adult stages, as well as the limited efficacy or high labor demand of alternative physical methods [4,5,6], have favored the use of biological control methods based primarily on entomopathogenic fungi, particularly of *Beauveria brongniartii* (Sacc.) Petch. strains [7,8] or, less frequently, with the more generalist species *Beauveria bassiana* (Bals.-Criv.) Vuill., whose endophytic characteristics open new perspectives for biocontrol strategies [9].

The level of efficacy achieved by applying biocontrol agents to control soil pests depends on several factors, particularly the virulence of the strain [10], the environmental conditions and formulation of the bio-inoculum, and the method of its application [11]. In the case of *Beauveria* species, the effect of environmental conditions, particularly the soil chemical characteristics and soil water status, have been demonstrated [12]. However, an additional element of variability for the efficacy of the bio-inoculum might derive from the biological fertility of the soil, particularly when managed according to organic farming practices [13,14]. Formulation of the bio-inoculum is critical because it is expected to support the strain’s competition with the better-adapted autochthonous soil microflora and to ensure a source of living cells able to interact with the pest [15,16]. Furthermore, the soil concentrations and persistence after soil applications of Beauveria species can be extremely variable, and depend on land use, climate, soil, and many other factors [17].

The production of the bio-inoculum can be further complicated by considering either that substrates composition, particularly of carbohydrates, can affect fungal virulence [10,18] or that the induction of assimilatory pathways by exogenous substrates, including lipid growth substrates, can have significant effects on the virulence of fungal infectious propagules [19]. The metabolism of the fungal strain is also crucial for successful infection to occur, as entomopathogenic fungi penetrate and dissolve the host-insect cell-wall during the mycoparasitic attack using chitinolytic enzymes [20,21], which can be used as markers for fungal activity against host insects. 

The authorization process for using microbial inocula as biopesticides in the Euro-pean Union requires evaluating the population background levels and the possibilities for survival, colonization, reproduction, and dispersal of the biopesticide, to avoid the risk of having adverse effects on other organisms in the natural environment. Indeed, the efficacy of entomopathogenic fungi is due to a wide range of metabolites, mostly secondary metabolism products, which can either act specifically against the host insect or be more general substances produced under a wide range of conditions. It should be considered that the range of host organisms of entomopathogenic fungi can be extensive and go beyond the subphylum boundaries. For example, *Beauveria bassiana* is capable of parasitizing several species of insects (Hexapoda), mites (Chelicerata), and millipedes (Myriapoda). Therefore, these metabolites, including toxins, also have different functions depending on the organism’s ecological niche at a given time and can also be modified in their structure and their chemical and biological functionality by natural processes in the soil [22]. It derives that the potential impact of these metabolites on non-target organisms can be a concern, for which European Union regulators require a specific assessment for the registration of potential biopesticides [23]. Therefore, the assessment of the impact of introduced bioinoculum on the soil microbial biodiversity, as well as its persistence, become critical. Still, it is also essential to support the understanding of the mechanisms of biocontrol and to interpret the results of efficacy tests. Such analysis would be even more important when inocula with multifunctional properties or consortia of strains are tested [24,25] and also considering the new “holobiont” concept about the intergenomic associations of the plant-pest-bioinoculum relation [26]. Indeed, understanding how the inoculants may affect the soil ecosystem as well as soil key ecosystem service, remains a major challenge.

The objective of this study, carried out to better characterize and understand the mechanism underlying the capacity of two strains of different *Beauveria* species, individually or as co-inoculants, to control *Melolontha* spp. grubs, was to evaluate their behavior in organic strawberry plantations in relation to the environmental conditions, their abundance after soil inoculation, their impact on soil microbial communities, and their in vitro chitinolytic activity with different carbon sources.

## 2. Materials and Methods

### 2.1. Field Trials

Two trials were carried out in strawberry plantations, managed according to organic farming practices, located in the territory of Lubartów district (Lublin voivodeship, southeastern Poland), namely at Brzostówka (51.4365° N, 22.7856° E) (henceforth, BZ) and Nowa Wola (51.4177° N, 22.7238° E) (henceforth, NW). Strawberry plants were planted in springtime (NW, cv. Polka) or late summer (BZ, cv. Senga Sengana) of 2014. Both sites had a similar soil texture (sandy loam), classified as podsolic, but different pH value (5.3 and 6.8, for BZ and NW, respectively), salinity (0.7 and 1.0 g NaCl · l^−1^ for BZ and NW, respectively) and organic matter content (1.06 and 1.19% on dry soil weight for BZ and NW, respectively).

Both fields (about 1 ha) were highly infested by *M. melolontha* larvae, as determined by an initial assessment of their presence made by counting live grubs before planting the strawberries (on average 2 larvae m^−2^ were found, 4-fold the acceptable damage threshold, i.e., 0.5 larva m^−2^). The assessment was performed collecting soil samples from 25 cm × 25 cm × 30 cm (w:l:d) wells and checking for the presence of the grubs (minimum 8 holes from each repetition).

A randomized blocks design with 4 replicates (for a total of about 1500 m^2^ per treatment) was established for the following treatments in both trials:

(1) A *Beauveria bassiana* strain (BB59, hereafter BA) isolated from rhizospheric soil of an apple orchard located in Valle d’Aosta by the company CCS Aosta, (Aosta, Italy), which genomic sequence of ITS region of the ribosome has been deposited in the GenBank database and can be accessed to ID KT932307. The strain is not registered for use as plant protection product.
(2) A *Beaveria brongniartii* strain (hereafter BR) isolated from the soil of a potato field highly infested by *M. melolontha* in Romanów locality (Lublin voivodeship, Eastern Poland). The strain is deposited in the Fungal Collection of the Institute of Agriculture and Horticulture, Siedlce University of Natural Science and Humanities. The sequence of the ITS region of the ribosome has been deposited in the GenBank database and can be accessed to ID KT932309.
(3) A consortium of the two strains (BA + BR) applied as a mixture of the two single formulations.

Control plots did not receive the bioinocula. The strains have not been tested before in laboratory to assess their virulence in comparison to other strains. However, pot experiments carried out under control conditions confirmed that both were able to infect *M. melolontha* larvae (unpublished data).

Both bioinocula were prepared by growing the fungi in submersed conditions in a liquid medium based on malt extract and glucose. Blastospores were collected by concentrating the suspension via centrifugation, dried onto a carrier material made of a mixture of corn fibers and zeolite (1:10 w/w), and formulated as a wettable powder. The concentration of each of the two fungi in the inoculum was about 1·10^7^ spores·g^−1^. All treatments were applied as an aqueous suspension. The applications were carried out near the plants’ row. A sprayer with large diameter nozzles and fan-less, to reduce the risk of damaging the fungal cells, was used to apply the equivalent of about 2000 L ha^−1^ of the bioinocula water suspension. After each application, the soil was mixed on the surface with a light hand hoeing.

Each treatment consisted of a dose of 45 kg·ha^−1^ applied to the soil. When the two fungal species were applied together, a half dose of each single formulation was distributed with the same application schedule. In case of the trial NW, the dose was split into four applications in the first year (starting on 20 May), with monthly intervals. For the trial BZ, the dose was split into two applications with three weeks interval in the first year (starting on 30 July). For both trials, a single application was performed the following two years (mid-June and mid-May in 2015 and 2016, respectively).

### 2.2. Assessment of Treatment Efficacy

Plant health status was evaluated several times after application of the products by counting the number of plants damaged by the grubs’ feeding activity, i.e., showing wilting symptoms due to damage to the root system and verifying the presence of the grubs on them, thus using a 0 (healthy) 1 (damaged) score. The assessments were made each time on 100 plants per repetition. For the trial NW, the first evaluation was performed at the end of the first growing season (September 2014), then twice in 2015 (June and October), and before the plantation was removed (July 2016). For the BZ trial, since the plantation was established in late summer 2014, the evaluation was performed twice in 2015 (June and October), and before the plantation was removed (July 2016). In spring (for trial NW) or autumn (trial BZ) of the second year (2015), damaged plants were replaced with new plants.

### 2.3. Assessment of the Presence of Entomopathogenic Fungi in the Soil

Soil samples were collected each year in the vicinity of the plants’ root system with an Egner’s sampler from a depth of 0–20 cm from about 25 points randomly distributed on each of the four plots for every treatment. These individual samples were merged to compose a laboratory sample (approximate weight 1–1.5 kg). The concentration of colony forming units (CFUs) of entomopathogenic fungi was determined using the selective medium developed by Strasser et al. [10]. Briefly, 2 g of soil were added to 18 mL of distilled water with an addition of 0.05% Trithon X-100 and vigorously shaken for about 60 s. An amount of 0.1 mL of the soil solution was poured and spread on the selective medium and incubated for 10–12 days at 22 °C, after which colonies were counted. A total of 16 repetitions per treatment (four laboratory repetitions per plot sample) were prepared for each sampling date. The species were determined by growing the pure cultures on Sabouraud (SDA) medium and then through microscopic analysis using standard keys or other relevant literature [27,28]. Given that only morphological methods were applied during the identification of fungi, the species *Beauveria bassiana* was defined sensu lato, because, as demonstrated by the latest phylogenetic studies based on DNA sequence [27,29] there are numerous fungal species within the genus *Beauveria*, which can be distinguished only by means of molecular markers. The total number of colonies of *B. bassiana* and *B. brongniartii* was determined as the number of colony-forming units (CFUs) per gram of dry weight of soil.

### 2.4. Analysis of Beauveria Strains Chitinolytic Activity and Metabolic Profile

The chitinolytic activity of the strains of the two *Beauveria* species alone and co-inoculated was studied in vitro, also assessing the ability of different carbon sources to elicit the synthesis of b-N-acetylglucosaminidase (NAGase) activity. A method combining the growth of the fungi in Biolog Phenotype MicroArray plates with the measure of NAGase activity, based on a chitinase fluorogenic substrate, was designed integrating the protocols developed by Niemi and Vepsäläinen [30] and Seidl et al. [31] as following specified.

The inoculation procedure for pure cultures of the *Beauveria* strains in the 96-wells FF MicroPlate^TM^ (Biolog Inc., Hayward, CA, USA), which contains 95 different carbon sources (https://www.biolog.com) plus water [32], was based on the original manufacturer’s supplied protocol and the protocol used by Canfora et al. [33]. For inoculum preparation, conidia of the two fungal strains were obtained by cultivation on Czapek agar plates (Oxoid) in the dark at 25 °C for 7 days. For inoculation, a sterile cotton wool swab was previously moistened in Biolog FF inoculating fluid (0.25% Phytagel, 0.03% Tween 40 in distilled water) and rolled over sporulating areas of the plates. The spores were suspended in sterile Biolog FF inoculating fluid and adjusted to an optical transmission of 75% at 750 nm (using a Biolog standard turbidimeter, calibrated to the Biolog standard for filamentous fungi, and FF inoculating fluid). The same suspension was used to inoculate the microplates either with the single strain inoculum or the co-inoculum (three biological replicates each). The microplates were inoculated with 100 μL of inoculum per well and incubated in the dark at 25 °C. A microplate reader at 490 nm (mitochondrial activity) [34] was used to read the microplates immediately after inoculation (time 0, as background plate value), then at 72 and 96 h of incubation.

After 96 h of incubation, the inoculated microplates were used to measure NAGase activity. An amount of 50 µL of 1 mM 4-methylumbelliferone-N-acetyl b-D-glucosaminide (Sigma Aldrich, St. Louis, MI, USA, code 69585) dissolved in sodium acetate buffer (acetate-buffer solution pH 4.6, Sigma Aldrich, code 31048) was added to each well of the microplates, which were incubated in the dark for 1 h at 30 °C with gentle agitation. An amount of 10 µL of the fluorogenic substrate-containing liquid from each well was transferred in as many wells of a black 96-well plate where 90 µL of sodium carbonate buffer pH 10 (Sigma Aldrich code S4132) was previously pipetted. The high pH of the carbonate buffer stops the enzymatic reaction and releases the 4-methylumbelliferone (7-hydroxy-4-methylcoumarin) (4-MUF) fluorochrome. These plates were immediately read in a fluorescence reader (Promega, GloMax^®^-Multi+, Madison, WI, USA), using an excitation wavelength of 355 nm and an emission wavelength of 460 nm [30]. The blank of the black plates consisted of a series of wells containing the carbonate buffer with the fluorogenic substrate in acetate buffer, not incubated with the fungi. The fluorescence values, after subtraction of the blank, were compared with standard curves obtained with standard solutions of 4-methylumbelliferone sodic salt. The values were reported as relative fluorescence units (RFU). All the solutions used in the measurement of NAGase activity were sterile, and the 4-methylumbelliferone-N-acetyl b-D-glucosaminide solution was filter-sterilized before its use.

### 2.5. Terminal Restriction Fragment Length Polymorphism (TRFLP) Analysis of Soil Microbial Community Structure and Diversity

The soil samples used for microbiological analyses were also used for the molecular analyses utilizing TRFLP analysis, which combines PCR, restriction enzyme digestion, and electrophoresis on automated sequencer to allow the detection of a single restriction fragment and surveying strains comprising at least 1% of the total microbial community [35].

DNA was extracted from 0.6 g of soil using the DNeasyPowerSoil^®^ DNA Isolation Kit (Qiagen Inc., Chatsworth, CA, USA) following the manufacturer’s instructions. DNA crude extracts yields were calculated using Qubit^®^ 2.0 Fluorometer (Thermo Fisher Scientific, Waltham, MA, USA), following the manufacturer’s instructions. DNA extraction was repeated in duplicates, and then the DNA solutions were pooled. The extracted DNA was diluted to 10 ng μL^−1^ and stored at −20 °C for the following analytical steps.

Primers 63f and 1087r labeled with VIC were used for the amplification of the bacterial 16S rRNA gene [36], whilst operon ITS1 and ITS4, labeled with fluorescent dye FAM (6-Carboxyfluorescein), were used for the amplification of the fungal ITS region of the ribosome (primers sequence details are provided in Supplementary Materials Table S1). PCR reactions were repeated in triplicate for each sample and were performed in a 30 μL volume with 50 ng of template DNA and 0.2 U of Platinum *Taq* hot start DNA Polymerase (Invitrogen, Waltham, MA, USA). The PCR was performed under the following conditions: 95 °C for 5 min followed by 30 cycles of 95 °C for 30 s, 55 °C for 30 s, and 72 °C for 1 min; the process was completed with a final extension step of 10 min at 72 °C. The PCR products were separated on a 1.5% agarose.

The amplified products were purified with a Qiaquick PCR purification kit (Qiagen Inc., Chatsworth, CA, USA), and 600 ng of amplified 16S rDNA was digested with 20 U of *TaqI* or *AluI* (Promega Inc., Madison, WI, USA) for 5 h at 37 °C and 65 °C, respectively. For the ITS region of the fungal DNA, 600 ng of the amplified product were digested with 20U of *HinfI* or *HaeIII* (Promega Inc., Madison, WI, USA) for 5 h at 37 °C. A 600 ng aliquot of the digested products was resolved by capillary electrophoresis on an ABI3500 Genetic Analyzer (Applied Biosystems, CA, USA) using LIZ600 as size standard for GeneScan analysis. Fragment sizes from 55 to 500 bp were considered for profile analysis and determination of the operational taxonomic units (OTU) numbers.

### 2.6. Quantification of Fungal Gene Copies

Quantitative PCR targeting specific taxonomic groups was used to monitor the soil fungal community status after inoculation and data used together with TRFLP analysis. Fungal DNA was amplified by means of the 5.8S/ITS1f primer pair [37] (primers sequence details are provided in Supplementary Materials Table S1). All qPCR reactions were carried out in 25 μL reactions containing 10 μL of template DNA (10 ng μL^−1^), 12.5 μL of Quanti Fast Sybr Green PCR Master Mix (Qiagen Inc., Chatsworth, CA, USA), 1.2 μM of primer, and PCR-grade water up to 15 μL. The reactions were performed in a Stratagene Mx3000P qPCR instrument (Agilent Technologies Inc., Santa Clara, CA, USA) and results were processed using the instrument software. Experiments were performed in duplicate. The qPCR of fungal sequences was carried out with an initial denaturation at 95 °C for 5 min, followed by 40 cycles of 95 °C for 1 min, 53 °C annealing for 30 s, and 72 °C extension for 30 s.

The absence of primers’ dimers in the amplification products was evaluated analyzing the melting curves of the products considering the fluorescence range at 50–99 °C. Moreover, PCR products were screened for purity and molecular weight in 1% agarose gel. The amplified products were purified after the qPCR reaction, quantified by Qubit^®^ 2.0 Fluorometer kit, following the manufacturer’s instructions, and diluted to minimize PCR bias [38].

The target *gene copy number* was calculated using the following formula, where *ng* is the amount of DNA, and *bp* is the template length:

$$gene\;copy\;number=\frac{\left(ng\times \frac{number}{mole}\right)}{(bp\;\times ng\;\left(g\;\times g\;mole\;of\;bp\right)}$$

(http://cels.uri.edu/gsc/cndna.html) [39].

Standard curves were created, for each targeted gene, amplifying the DNA of soil samples mixture from each experimental field trials (representing all treatments) and calculating the target gene copy number. Standards were obtained in triplicate using a 10-fold dilution series, covering six orders of magnitude from 10^2^ to 10^8^ gene copies per qPCR reaction during each run. Target copy numbers for each reaction were calculated from the standard curves. 

Fungal copy numbers were expressed per gram of dry soil weight, and the relative values were log-transformed. The mycelia of the two strains (BA and BR) grown on solid agar media were used for fungal quality control of amplification products.

### 2.7. Data Treatment and Statistical Analyses

#### 2.7.1. Field Trials and Microbiological Analysis of Soil Samples

Field data were analyzed by ANOVA using R package version 3.6.1 [40] considering the treatment and the season as factors. Means were separated by Tukey test at *p* < 0.05 significance level. Percentages from the assessment of damaged plants were preliminarily subjected to Bliss’s transformation to comply with statistical assumptions of ANOVA. Efficacy of the treatments was calculated according to Abbott formula [41]. Data from microbial analyses were analyzed by ANOVA, separating the means by Tukey test at *p* < 0.05 significance level.

#### 2.7.2. Analysis of the Interactions between Climatic Conditions, Entomopathogenic Fungi Abundance in Soil and Plant Damage

Monthly air temperature and humidity, together with the monthly sum of precipitation, were collected from a weather monitoring station (Lublin Radawiec) of the Institute of Meteorology and Water Management closely located to the field sites. These parameters, along with the data of abundance of *B. bassiana* and *B. brongniartii* in soil were utilized to evaluate their effect on the rate of damaged plants. The stats package from R package version 3.6.1 [40] was used to perform a principal component analysis using the *prcomp* function and the Pearson correlation coefficient was calculated using the *cor* function. The plots were generated with *ggbiplot* function using ggplot2 [42].

#### 2.7.3. Analysis of Biolog and NAGase Activity

Data obtained from the Phenotype MicroArray^TM^ (Biolog, Hayward, CA, USA) assays were used to compare the substrate use by the two fungal species and their co-inoculum with the corresponding NAGase activity. One-way ANOVA was performed using XLSTAT, 2019.1.3 software [43]. ANOVA was followed by Tukey’s HSD (honestly significant difference) post hoc test. The absorbance at 490 nm of the time zero sample for each plate was subtracted from readings, while the absorbance of the water-containing well was used as a variable in the analysis.

In order to evaluate the overall fungal metabolism and the ability of main categories of carbon sources to trigger the NAGase activity, the 95 substrates (96 including water used as “outgroup”) were classified into the following 16 functional groups [32,44]: heptose, hexoses, pentoses, sugar acids, hexosamines, polyols, polysaccharides, oligosaccharides, peptides, L-amino acids, biogenic and heterocyclic amines, TCA-cycle intermediates, aliphatic organic acids, and other compounds. The average absorbance and NAGase activity for all wells in each group were calculated. Heat maps were built to visualize both the substrate use and the chitinolytic activity of the two fungal strains and the co-inoculum. In the heat maps, prepared with *R* statistical software [40] using *Bioconductor* package, a clustering on both columns and rows of a features/individuals data matrix was applied. Both features and individuals were clustered independently using ascendant hierarchical clustering based on Euclidian distances. The data matrix’s rows and columns were then permuted according to the corresponding clusters. After scaling, the negative values were related to downregulation and positive to upregulation of the enzyme activity. In the heat maps displayed, data values were replaced by corresponding color intensities [45].

#### 2.7.4. Analysis of TRFLP Profiles to Assess Soil Diversity

A derivative profile was created by comparison of TRFLP profiles from each restriction enzyme from duplicate DNA samples, following the method reported by Canfora et al. [33,36]. The quality of TRFLP data was checked by GeneMarker software (SoftGenetics LLC, State College, PA, USA). Each peak on the TRFLP profiles is thought to correspond to a certain anonymous taxon referred to as OTU, whilst the area of the peak is thought to correspond to the proportion of this OTU in the microbial community. Only fragments with a fluorescence intensity of ≥55 arbitrary units of fluorescence were considered, and the total amount of DNA of each profile was calculated by summing the area of all peaks. Alignment of the profiles was performed directly on the output table of the software GenMarker, considering ±0.5 bp to discriminate peaks of consecutive sizes.

Diversity indexes analysis, calculated using the PAST program [46], was performed on derivative TRFLP profiles of the different enzymes that were combined together and transformed into a binary vector, in which the intensities area of peaks were scored as strings, to be used for the analysis. The Shannon–Wiener index (H’) was calculated as a measure of community profiling diversity [46], and the OTU number was used to evaluate the relative abundance of the microbial community. Results derived by TRFLP analysis carried out from the soil of the two investigated sites were compared using one-way ANOVA and Fisher’s post hoc statistic testing for significance level at *p* < 0.05 using XLSTAT software [43].

## 3. Results

### 3.1. Effect of Bioinocula on Melolontha spp. Damage of Strawberry Plants in Organic Plantations

**Figure 1:**
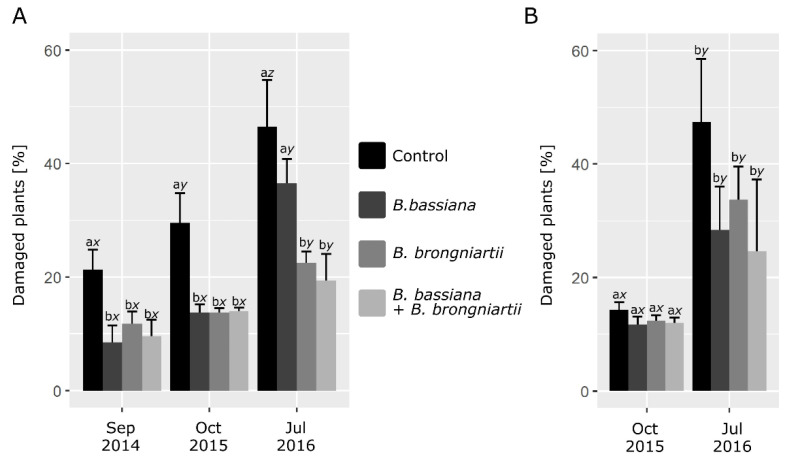
Effect of the application of formulated entomopathogenic fungi (*Beauveria bassiana* and *Beauveria brongniartii*) as a single inoculum or as mixture, on the number of damaged strawberry plants by grubs of *M. melolontha*. (**A**) trial NW (Nowa Wola) (**B**) trial BZ (Brzostówka). Means ± SD. Letters are showing the statistical differences between treatments within the same assessment period (a–c) and between periods within the same treatment (*x*–*z*) for *p* ≤ 0.05.

The application of the formulated inocula both as single strain or as a co-inoculum reduced the damage from grubs of *M. melolontha* only in one (NW) of the two plantations (Figure 1). The damage was about 50% in comparison to untreated control each year, irrespective of the strain used, with the exception of BA at the end of the third year. In both locations, the percentage of damage increased during the years, for both treated and untreated plants, but in general to a lesser extent for the plants growing on inoculated soil than in untreated ones. This has resulted in a significant interaction between the treatment and the season in the statistical analysis for the NW trial (*p* ≤ 0.001) and almost significant for BZ trial (*p* = 0.055) (Supplementary Material Table S2). Consequently, the efficacy of the applied bioinocula ranged considerably between the sites and the seasons (Table 1).

### 3.2. Monitoring of Beauveria Species in Soil

The soil analysis pointed out a different situation concerning the natural presence of *Beauveria* species in the two locations (Table 2). *B. bassiana* could be considered a natural entomopathogen of the two fields, as it was isolated from every untreated soil sample, regardless of the location of the plantation or the sampling time. On the contrary, *B. brongniartii* was never detected in the soil of the control treatment during the whole period of the trials in both locations, and was detected only after the application of the bioinocula containing the BR strain (both alone and as co-inoculum). A significant transient increase of the *B. bassiana* soil population compared to the control was observed only few weeks after the application of the bio-inoculum: in September 2014 at BZ site after the application on July-August, and in July 2015 in NW after the application in June (Table 2). Similarly, applying BR resulted in the detection of the species, though to a different extent depending on the site: higher in NW than in BZ with respect to the control. The abundance of the two fungal species was not consistently increased by the co-inoculum in both sites and during the different seasons.

### 3.3. Evaluation of the Interactions Between Climatic Conditions and Abundance of the Soil Entomopathogenic Fungi Populations on the Level of Plant Damage

**Figure 2:**
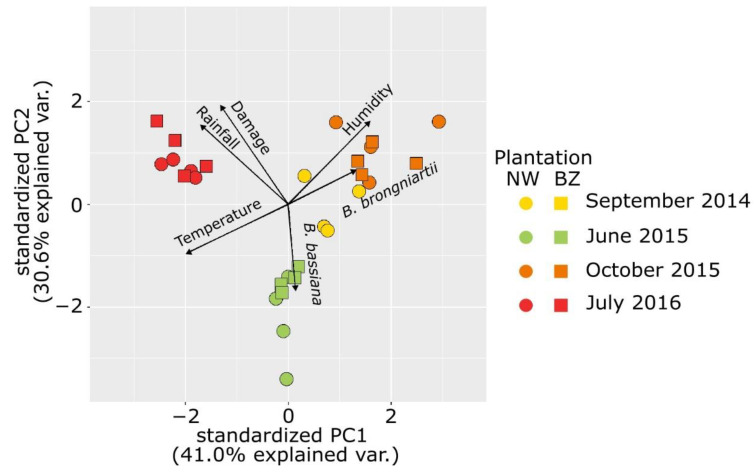
Principal component analysis of the relationship between environmental conditions, plant damage, and entomopathogen fungi abundance in the soil in the two strawberries trials during the three growing seasons.

The analysis of the interactions between climatic conditions (average monthly temperature and air humidity and monthly sum of rainfall), the degree of damaged plants, and the abundance of *B. bassiana* and *B. brongniartii* in the soil for both trial sites showed a clustering of the data into groups according to the season, regardless the location of the trial site (Figure 2 and full data set presented as Supplementary Material Table S3). It has been observed that changes in the climatic conditions between years were mainly recorded in the precipitations pattern, while very similar patterns of the temperatures were registered in both years during the growth season (Supplementary Material Table S3).

### 3.4. In Vitro Metabolic and Chitinolytic Activities of the Two Beauveria Strains

**Figure 3:**
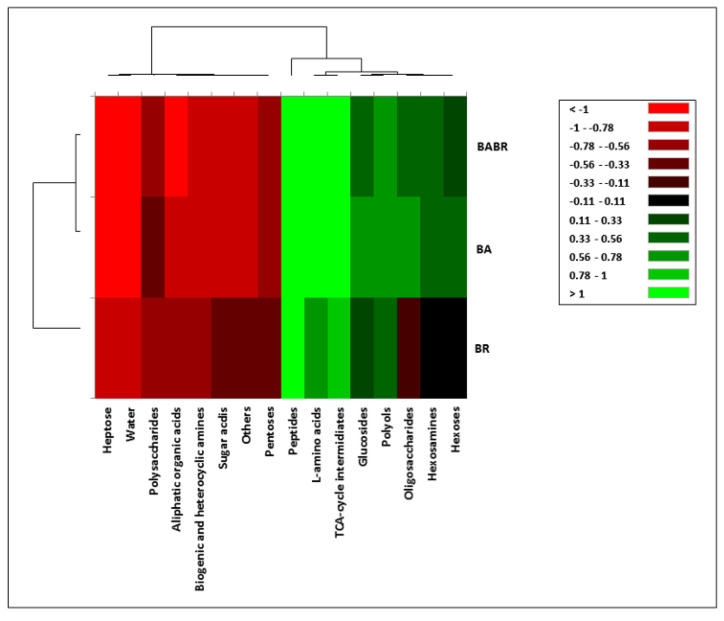
Heat map of in vitro carbon source use by the strains of *B. bassiana* (BA) and *B. brongniartii* (BR) or their co-inoculum (BA + BR) (490 nm readings after 96 h incubation).

The metabolic activity of *B. bassiana* was generally higher in all substrates in comparison to *B. brongniartii* (Figure 3, Supplementary Material Table S4). The co-inoculum of the two fungal strains showed a similarity of the carbon sources metabolism with that of BA, indicating the prevalence in metabolic efficiency of this species over BR (Figure 3, Supplementary Materials Table S5a,b). However, some substrates (e.g., L-aspartic acid, D-mannitol, L-alanine, the group of peptides) triggered the activity of the two species when co-inoculated, inducing a higher metabolism of the co-inoculum in comparison to that of the single inoculum (Supplementary Material Table S5a,b).

Two distinct groups of compounds could be discriminated according to their capacity of inducing a metabolic activity in the two species and the co-inoculum. One group, composed by peptides, L-amino acids, intermediates of the tricarboxylic acid cycle, glucosides, polyols, oligosaccharides, hexosamines, and hexoses, stimulated the metabolism of both species and their co-inoculum (green block on Figure 3, Supplementary Material Table S5b). The other group, containing heptoses, polysaccharides, aliphatic organic acids, biogenic amines, sugar acids, pentoses, other compounds, and water, was practically not metabolized by the two species and their co-inoculum (red block on Figure 3, Supplementary Material Table S5b). 

**Figure 4:**
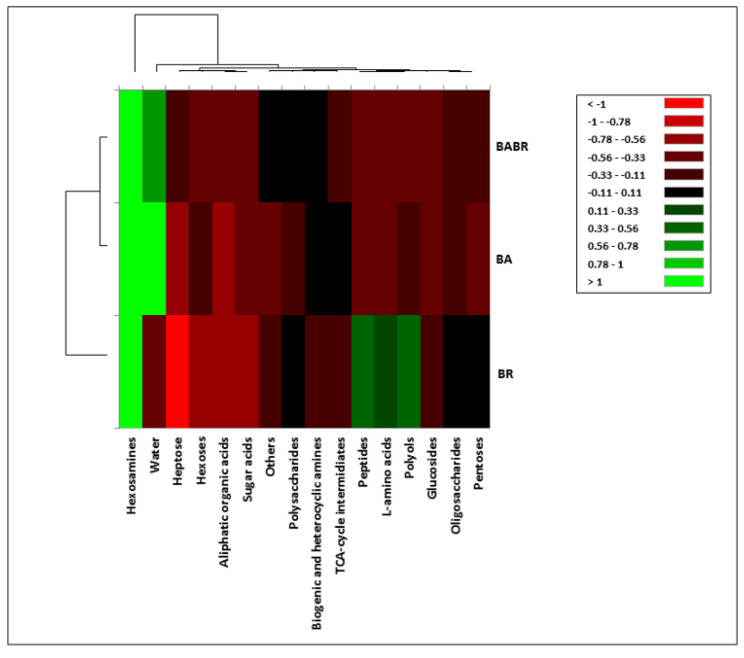
Heat map of NAGase activity by the strains of *B. bassiana* (BA) and *B. brongniartii* (BR) or their co-inoculum (BA+BR) (readings after 1 h incubation).

The chitinolytic activity of the two species alone and of the co-inoculum was differently stimulated by the diverse carbon sources (Figure 4 and Supplementary Material Table S6a,b). Specific substrates stimulated the chitinolytic activity of either one or the other species of *Beauveria* or the co-inoculum (e.g., N-acetyl-D-galactosamine, succinic acid monomethyl ester, b-cyclodextrin, D-glucuronic acid, sedoheptulosan, D-saccharin acid), with a different degree of intensity. Interestingly, the chitinolytic activity of BR, compared to BA and the co-inoculum, was significantly triggered by some specific substrates (e.g., D-cellobiose, dextrine, adonitol, i-erythritol, L-aspartic acid, D-ribose, amygdalin, D-sorbitol, etc.) (Supplementary Material Table S6a). D-glucosamine, N-acetyl-D-glucosamine, and N-acetyl-D-galactosamine induced the highest NAGase activity in both species and the co-inoculum (Supplementary Table S6a). However, only N-acetyl-D-galactosamine triggered a significantly different response between the two strains and their co-inoculum, with the latter statistically higher than in BA and BR alone; D-glucosamine and N-acetyl-D-glucosamine were instead used as a carbon source by both the single inocula and the co-inoculum in a similar way (Supplementary Materials Table S6a). 

When considering the functional groups of substrates, the chitinolytic activity was significantly higher in the co-inoculum grown at the presence of hexosamines (N-acetyl-D-mannosamine, N-acetyl-D-galactosamine, N-acetyl-D-glucosamine, and D-glucosamine) (Figure 4, Supplementary Materials Table S6b). Chitinolytic activity in BR was significantly higher than in BA and the co-inoculum when grown on peptides, polyols, and L-amino acids (Figure 4, Supplementary Materials Table S6b). Moreover, when BA was grown without carbon sources (water), corresponding to a condition of oligotrophy and stress, it showed higher chitinolytic activity compared to BR and the co-inoculum (Figure 4, Supplementary Materials Table S6a,b).

### 3.5. Impact of Bioinocula on Soil Biodiversity

To assess the effects of the single inoculated strains and their co-inoculum on soil autochthonous microbial populations, we analyzed the soil bacterial and fungal community structure applying a TRFLP-based methodology capable of surveying members comprising at least 1% of the total community and also quantifying the gene copy numbers of a specific set of genes. Based on the analysis of TRFLPs, the application of the bioinocula did not alter considerably or consistently the composition of both fungal and bacterial communities neither after the first application nor at the end of the second season in comparison to untreated soils (Table 3). OTUs number, and consequently the Shannon-Wiener diversity index (H’), of fungi were higher after application of BA in 2014 in both sites, but a slight decrease was observed in 2015. Similarly, OTUs and H’ index varied for bacteria after application of BR, but with opposite trends in the two sites.

These results were also confirmed when utilizing the analysis of gene copy number and evaluating the ratio of the two fungi on the total fungal population (Table 4, Supplementary Materials Table S7). In this case, an increase (one order of magnitude) of the inoculated species was found for *B. brongniartii* in both trials in 2015, but confirmed only in the BZ trial in the following year. 

## 4. Discussion

### 4.1. Efficacy of Bioinocula in the Control of Melolontha spp. Damage and in Relation to Environmental Conditions

The trials were established to verify the effect of a treatment based on a generalist entomopathogenic fungus (*B. bassiana*), which is adaptable to different environmental conditions and able to live as saprophyte and endophyte, in comparison to the use of a specific parasite (*B. brongniartii*), which has been applied mainly against *Melolontha* spp. and to verify whether their co-inoculum could improve the overall efficacy of the treatment benefiting from their different behavior. A reduction in the number of damaged plants was observed when compared to the untreated control only in one location, with a sufficient efficacy according to organic farming standards, irrespective of the strain used and whether applied as single or as co-inoculum. On the other hand, the application of bioinocula resulted only in a transient increase in the abundance of both species lasting for some weeks after the distribution of the bioinocula, generally higher when the single strain was applied, as it could be expected considering that the dose of each co-inoculated strain was half of that used for each single inoculum. Insufficient fungal density after the application of the bioinoculum or its survival capacity are deemed the most critical factors affecting the efficacy of *Beauveria* strains against *Melolontha* spp. grubs [47]. According to Enkerli et al. [48], effective control of the grubs can be achieved when fungal density in soil reaches 1 × 10^3^–1 × 10^4^ CFUg^−1^. Densities at this level for both *Beauveria* species were not found so frequently in our experimental sites. A positive correlation between *B. brongniartii* density in soil and *M. melolontha* mortality during trials using a particularly virulent strain (BIPESCO 2) was found on potato fields [7]. This confirmed the finding of Kessler et al. [12] that the reproduction of *B. brongniartii* in the soil mainly depends on the presence of *M. melolontha* grubs. The average incidence of grubs in the strawberry plantations at the beginning of the trials described here was four times the threshold, but, even though very damaging for the crop, not reaching levels of incidence found in other trials [8,49]. However, the high variability in the belowground infestation levels on the local scale, somehow related to the soil characteristics, have also been proposed as an explanation for the different efficacy observed in the field in controlling soil-borne pests [50]. It is also possible that the pathogenicity of the tested strains was lower than that of strains used in other reports or that the production and formulation process negatively affected their virulence [10,18] since the same BR strain formulated as kernels was found to be more effective in other trials [51].

The environmental conditions of the two sites seemed to be non-conducive for *B. brongniartii* as indicated by the absence of its detection with the microbiological method in untreated plots, confirming its relatively low abundance in the area [52]. Such condition could have also affected its pathogenicity, since entomopathogenic fungi strains differ in their “ecological fitness” i.e., ability to persist in the field and successfully infect a host under sub-optimal conditions [53]. Nevertheless, we did not observe a relation between the abundance of both strains and the number of damaged plants. However, since the density of *B. bassiana* in soil was a sum of the native *B. bassiana* population (always detected also in control plots) and the applied strain, the higher efficacy observed in plots receiving the bio-inoculum in comparison to untreated ones could be the result of a higher virulence of the bio-inoculum towards *Melolontha* spp. grubs than the autochthonous entomopathogenic fungal population [15]. This interpretation is also supported by the fact that a significant decrease in plant damage after *B. bassiana* treatment in comparison to control was found even when there were no significant changes in the *B. bassiana* abundance in soil. It is noteworthy that, in both locations, BA was able to reduce the damage to a level similar to the specialized BR. Aside from the effect of pedo-climatic factors (see below), the broader metabolic capacity of the BA strain than BR emerged in the Biolog experiments could also account for such result.

Pedo-climatic conditions can affect the efficacy of *Beauveria* spp. bioinocula [54] and this emerged clearly from the analysis of the interactions between the level of plant damage from *Melolontha* spp. grubs and the abundance of entomopathogenic fungi populations in the soil, which showed a strong relation to climatic conditions, particularly the soil humidity as derived from the recorded precipitations pattern, irrespective of the location of the trial. Interestingly, the soil samples collected in autumn resulted in the highest abundance in *B. brongniartii*, while those collected in the periods with high temperatures and lower humidity (June) showed higher abundance of *B. bassiana.* This species presents a slightly higher optimum temperature for growth (23–28 °C) than *B. brongniartii* (22–23 °C) [54], which was also reflected in the negative correlation between *B. brongniartii* density in soil and the recorded temperature emerged from the analysis. A reduced efficacy after application of *Bassiana* spp. was observed in sandy soils [55], i.e., with a texture similar to that of the trials fields, or in case of insufficient soil moisture [56]. The different persistence and efficacy found in the two trials could be accounted for also by the chemical characteristics of the soil, particularly the pH (more acidic in the BZ field than in NW) and the organic matter content (slightly lower in the BZ field than in NW). According to Karthikeyan et al. [57] the optimal soil pH for *Beauveria* spp. development ranges from 6 to 8. However, Quesada-Moraga et al. [58] found that the occurrence of *B. bassiana* was correlated with the pH of soil, being more frequent at a narrow pH range (52.9% occurrence at the 8–8.5 pH level). Assessing the tolerance and optimum pH ranges in 29 isolates of *B. bassiana* derived from different insect sources and locations world-wide, Padmavathi et al. [59] found that all isolates tolerated a pH of 5–13, with few of them having tolerance to lower (4) and/or higher (14) pH as well. However, only sixteen of them showed a wide pH optimum. Furthermore, acidic soils, with a pH similar to that of BZ site, were found to sharply reduce *B. brongniartii* capacity of grubs infection [60], while acidic pH was found to be optimal for toxin production in *B. bassiana* [61]. The resilience of agricultural soils, particularly those managed for an extended period according to organic farming methods as those concerned by the trials, with regard to the introduction of bioinocula could also represent an essential factor to be taken into consideration when evaluating the efficacy of bioinocula [52]. The different plant genome of the two strawberry cultivars grown in the trials could also be an additional factor contributing to the observed different efficacy of the bioinocula [62,63].

The hypothesis of achieving a higher efficacy by combining the two species was not confirmed by the trials, likely as a result of the different factors affecting the single strain with the additional effect of a lower abundance, particularly for BR, eventually associated to the BA metabolic competitive advantage emerged from the phenotypic characterization of the co-inoculum (see below). The combination of different species or strains for biocontrol purposes has been utilized mainly for diseases control [64], while in case of entomopathogenic fungi it is still an underexplored approach. A synergistic activity in the pathogenicity against *Duponchelia fovealis* of consortia composed by two fungi using strains of different species (*B. bassiana*, *Purpureocillium lilacinum* and *Isaria javanica*) was reported and found to be also associated to increased chitinase and lipase activities [65]. Nevertheless, consortia formed by two strains of *B. bassiana* (with one of them being the same strain in both consortia) induced either a lower or a higher *D. fovealis* mortality than the two strains alone, thus showing that both synergistic and antagonistic effects can occur.

### 4.2. Impact of the Bioinocula on Population Abundance of Beauveria Species and Microbial Biodiversity

According to the microbiological evaluation of the soil population of the two fungal species, *B. bassiana* could be considered a common native species in the studied locations, while *B. brongniartii* was never found in the untreated soil samples. This result is in line with other reports showing that *B. bassiana* was a dominant entomopathogenic fungal species under different soil conditions [66,67,68,69,70,71]. However, its detection, as well as for other entomopathogenic fungi, can be affected by the method used for the analysis [70]. Considering that *B. bassiana* is the most widely distributed species of the whole *Beauveria* genus, with a wide range of parasitized species and capable of living as an endosymbiont of several plants species, its survival capacity and adaptation plasticity is not surprising [54,58]. On the other hand, the limited frequency of *B. brongniartii* even after the application of the BR product is likely derived from its host specificity (*Melolontha* spp.) under natural conditions in Central Europe [12,72] and not favorable pedo-climatic conditions [73] of the studied sites.

The application of the fungal bioinocula individually or as co-inoculum did not consistently alter the natural microbial biodiversity in the soil environment as expressed by the OTU number and Shannon–Wiener diversity index. This confirms the results of a monitoring of fungal diversity in agricultural soils treated with *B. bassiana* [74] or *B. brongniartii* [75] and from the analysis of the structure and functional diversity of microorganisms within the rhizosphere of maize after *B. bassiana* application [76].

The analysis of the overall abundance of the two inoculated species on fungal community based on molecular data was utilized to evaluate the impact of the two bio-inoculants and their co-inoculum on soil biodiversity. When such level of investigation was used, a small effect of the application of the bioinocula containing *B. brongniartii* on the fungal community appeared after the third year of treatment. The technique based on TRFLP has been applied to examine microbial community structure and community dynamics in response to changes in different environmental parameters, or, to study fungal population composition in natural habitats [35,77]. However, TRFLPs provide only a qualitative result. Therefore, the qPCR (quantitative polymerase-chain-reaction) was utilized in the study to evaluate the abundance of genes typical of fungi. Different reasons could be hypothesized to explain the observed changes. The inoculative effect may be a consequence of the seasonal variations [78] or modification of the trophic populations of nematodes [79] that are inducing fluctuations in the community structure without a disturbance that alters the overall soil biodiversity. This effect could even promote potential synergistic interaction within soil microbial community [80]. On the other hand, the effect could be derived by the cultivation-independent methods used and the relevant bias or critical steps intrinsic to these methods [81], the results of which could be relatively insensitive to species richness thus not reflecting shifts in relative abundance of species, with consequences for the evaluation of the impact on biodiversity. Related to this issue is the need to develop tools for tracing and monitoring the introduced species in soil to address their persistence and their fate, which indirectly provide important insights into the overall effects on soil microbial ecology [39]. Nevertheless, it is noteworthy that only *B. brongniartii*, not being resulted a common native species of the studied sites from the microbiological analyses, has transiently modified its share in the total fungal population. It could also be observed that such effect, noticed particularly in the soil from BZ trial, could be linked to the efficacy of the treatments in reducing the damage from *M. melolontha* grubs: even though statistically not significant, due to the high variability of the data, the BR inoculum allowed to limit the damage in the second year.

### 4.3. In Vitro Metabolism and Chitinolytic Activity as a Tool to Assess Bioinocula Potential

The phenotypic analysis using 95 different carbon sources showed that the *B. bassiana* strain exhibited broader catabolic properties and a faster growth rate than the *B. brongniartii* strain. However, only a group of substrates resulted in being promptly utilized, also when the two strains were co-inoculated. The higher metabolic capacity of *B. bassiana* over *B. brongniartii* could derive from the broader range of host species of *B. bassiana* than *B. brongniartii* living behavior [54]. Differences in the metabolic pattern between three *B. bassiana* and five *B. brongniartii* strains were pointed out, allowing to discriminate them into two separate groups [82]. However, the combined application of more than one strain as bio-inoculum could lead to various interactions between the used strains (competition or cooperation) [83], thus potentially influencing the efficacy of the application of mixed bioinocula as tested in the field trials. This interaction was confirmed by the different metabolic profile showed by the co-inoculum compared to the single strains as emerged from the in vitro experiments, even though the partial overlap between the co-inoculum and BA profiles suggests a form of interaction between the two fungal species in relation to their different ecological niches [84] since niche overlap is a key factor in species coexistence [85].

The analysis of the chitinolytic activity was performed to evaluate the ability of individual substrates to stimulate chitinolytic activity as a marker of strain virulence against pests. Indeed, entomopathogenic fungi such as *Beauveria* species produce multiple extracellular enzymes, including chitinolytic and proteolytic enzymes, that facilitate host infection [86]. Two compounds, D-glucosamine and N-acetyl-D-glucosamine, stimulated the fungal NAGase activity to the greatest extent. This was expected, since N-acetylglucosamine (GlcNAc), an amide derivative of glucose, is the monomeric unit of the chitin and glucosamine is the precursor of biochemical synthesis of glycosylated proteins and lipids and part of the structure of chitosan and chitin [86]. However, it was found that other substrates were also capable of inducing this activity in the fungi (e.g., other compounds belonging to hexosamines). It is noteworthy that hexosamines triggered the chitinolytic enzyme production despite the fact that they were not among the carbon substrates that highly stimulated the development of the fungi.

Interestingly, some substrates were triggering NAGase activity on a species-specific base and this also holds for the co-inoculum, which expressed more NAGase on particular carbon sources (e.g., N-acetyl-D-galactosamine). Chitinase gene expression in entomopathogenic fungi is believed to be controlled by a repressor-inducer system in which chitin or the oligomeric products of degradation serve as inducers [87]. Furthermore, fungal chitinases are also capable of morphogenetic functions and defense against other organisms located in the same ecological niche [86,88], which could also explain the different enzymatic activity between the single strains and the co-inoculum. The discrepancy observed for some carbon sources between mycelial development and the enzymatic expression may also support the results of the field trials for what concerns the efficacy of the treatment and abundance of the two strains and be useful for the development of a formulation of mixtures of fungi and carbon sources to increase the pest control efficacy.

## 5. Conclusions

In this study, the efficacy of two strains of *B. bassiana* and *B. brongniartii* applied as a single inoculum or co-inoculum to control *Melolontha* spp. grubs on organic strawberry plantations was compared, trying to relate it to the pedo-climatic conditions, the fungi metabolic activity and also assessing their impact on the soil microbial biodiversity. Regardless of the abundance of both *Beauveria* species in soil, it was possible to observe a certain reduction in the number of plants damaged by *Melolontha* spp. grubs, even though the efficacy appeared to be affected by soil and seasonal conditions. Other possible factors influencing the strains’ capacity to control the grubs have been hypothesized and should be taken into consideration when applying bioinocula, particularly in organically managed crops. The *B. bassiana* strain showed a higher metabolic versatility in the use of different carbon sources in vitro compared to the *B. brongniartii* strain, whose profile was partly overlapped in the co-inoculum. This behavior and the significant differences in the chitinolytic activity of the strains and of the co-inoculum could also be related to the efficacy of pest damage control. The strong in vitro metabolic activity of the *B. bassiana* could be associated to the higher abundance of this species in the soils receiving this strain in comparison to those receiving the *B. brongniartii* one, regardless it was applied as a single inoculum or as co-inoculum. On the other hand, a natural population of *B. brongniartii* was never detected in the studied sites and generally the applied strain persisted less in the soil after the treatment, showing a lower capacity to adapt to the pedo-climatic conditions of the trials’ sites than the *B. bassiana* strain. Neither used strain was consistently affecting the soil microbial (fungi and bacteria) biodiversity when this parameter was assessed using microbiological or DNA-based methods. A small transient increase of the share of the inoculated species to the total fungal community was noted only for *B. brongniartii* at the end of the third growing season when the analysis of genes copy numbers was used to this aim. This result underlines the need for a broader analysis to fully appraise the interactions between autochthonous soil microbiome and the bioinocula.

## Figures and Tables

**Table 1:** Efficacy of formulated products containing the entomopathogenic fungi (*Beauveria bassiana* (BA) and *Beauveria brongniartii* (BR)) as a single inoculum or as mixture (BA + BR), on reducing the damage to strawberry plants by grubs of *M. melolontha*.

Treatments	Efficacy (%) According to Abbott		
	September 2014	October 2015	July 2016
	Trial NW #		
Control	-	-	-
*B. bassiana*	60.1	53.2	21.5
*B. brongniartii*	44.6	53.2	51.6
BA + BR	54.9	52.5	58.5
	Trial BZ #		
Control	-	-	-
*B. bassiana*	-	18.2	30.5
*B. brongniartii*	-	14.0	17.2
BA + BR	-	16.1	23.1

# Plantation NW was established and started to be treated at spring 2014, while plantation BZ was established and started to be treated at the end of summer 2014.

**Table 2:** Number of colony-forming units (CFU) (10^3^g^−1^ soil) of two entomopathogenic fungi populations (*B. bassiana* and *B. brongniartii*) determined in soil by microbiological selective media method as affected by the application of the bio-inoculum of the two strains alone or co-inoculated (BA + BR). Means ± SD *.

Treatment	Species Determined (CFU 10^3^ g^−1^ Soil)							
	September 2014		July 2015		October 2015		July 2016	
	*B. bassiana*	*B. brongniartii*	*B. bassiana*	*B. brongniartii*	*B. bassiana*	*B. brongniartii*	*B. bassiana*	*B. brongniartii*
Trial NW #								
Control	0.33 ± 0.5	0.0 a	1.67 ± 1.7 a	0.0 a	0.33 ± 0.5	0.0 a	2.00 ± 0.8	0.0
*B. bassiana*	1.33 ± 0.5	0.0 a	4.67 ± 0.5 b	0.0 a	1.67 ± 0.9	0.67 ± 0.5 a	1.00 ± 0.1	0.0
*B. brongniartii*	1.00 ± 0.1	3.00 ± 0.8 b	0.67 ± 0.5 a	0.67 ± 0.5 b	0.67 ± 0.5	6.00 ± 1.4 b	0.33 ± 0.5	0.0
BA + BR	1.67 ± 0.9	0.33 ± 0.5 a	2.67 ± 1.2 a	0.0 a	0.33 ± 0.5	1.00 ± 0.8 a	0.33 ± 0.5	0.0
Trial BZ #								
Control	4.00 ± 0.8b	0.0 a	0.67 ± 0.5	0.0 a	0.67 ± 0.5	0.0 a	0.33 ± 0.5	0.0
*B. bassiana*	5.33 ± 0.5b	0.0 a	1.00 ± 0.8	0.0 a	1.00 ± 0.8	0.0 a	1.00 ± 0.1	0.0
*B. brongniartii*	2.00 ± 1.4a	1.00 ± 0.1 b	0.33 ± 0.5	1.33 ± 1.3 ab	0.0	1.00 ± 0.8 ab	0.0	0.0
BA + BR	1.67 ± 0.5a	0.0 a	0.67 ± 0.5	1.00 ± 0.8 b	1.67 ± 0.9	4.00 ± 0.1 b	0.67 ± 0.5	1.33 ± 0.5

* Values in column for each trial with different letters are significantly different for *p* ≤ 0.05. # In 2014, at the NW site four monthly treatments were applied starting from planting (20 May), while two applications (from planting on 30 July) with three weeks interval occurred at the BZ site. For both trials, a single application was performed the following two years (mid-June and mid-May in 2015 and 2016, respectively).

**Table 3:** Effect of application of the bioinoculum of the two strains of the entomopathogenic fungal species (*B. bassiana* and *B. brongniartii*) alone or co-inoculated (BA + BR) on OTU numbers and Shannon–Wiener diversity index calculated from TRFLS data of soil samples from the two trials.

Trial NW								
Treatment	September 2014				July 2015			
	Fungi		Bacteria		Fungi		Bacteria	
	OTU number	H’ index	OTU number	H’ index	OTU number	H’ index	OTU number	H’ index
Control	14	1.62	21	1.32	104	4.60	11	2.34
*B. bassiana*	29	1.90	15	1.29	95	4.50	13	2.52
*B. brongniartii*	17	1.82	35	2.15	115	4.70	6	1.65
BA + BR	ND *	ND	ND	ND	91	4.20	12	2.53
**Trial BZ**								
Treatment	September 2014				July 2015			
	Fungi		Bacteria		Fungi		Bacteria	
	OTU number	H’ index	OTU number	H’ index	OTU number	H’ index	OTU number	H’ index
Control	16	1.80	19	2.00	79	4.38	14	2.60
*B. bassiana*	23	2.60	23	1.90	58	4.12	9	2.49
*B. brongniartii*	11	0.90	5	1.10	47	3.84	10	2.30
BA + BR	ND	ND	ND	ND	82	4.40	10	2.30

* ND = Not determined.

**Table 4:** Share of two entomopathogenic fungal species (*B. bassiana* and *B. brongniartii*) after application of strains of these species alone or co-inoculated (BA + BR) on the total soil fungal population determined on the basis of gene copy number analysis.

Treatment	Trial NW							
	2015				2016			
	BA/total fungi		BR/total fungi		BA/total fungi		BR/total fungi	
	May	July	May	July	May	July	May	July
Control	3.36	3.60 × 10^−3^	5.18 × 10^−3^	7.49 × 10^−3^	3.04 × 10^−3^	1.78 × 10^−2^	5.15 × 10^−4^	1.83 × 10^−3^
*B. bassiana*	1.17 × 10^−2^	6.47 × 10^−3^	-	-	2.65 × 10^−3^	8.05 × 10^−3^	-	-
*B. brongniartii*	-	-	1.38 × 10^−2^	1.04 × 10^−2^	-	-	4.74 × 10^−4^	2.11 × 10^−3^
BA + BR	7.30 × 10^−3^	7.84 × 10^−3^	1.11 × 10^−2^	1.71 × 10^−2^	3.82 × 10^−3^	6.05 × 10^−3^	4.94 × 10^−4^	9.39 × 10^−4^
	**Trial BZ**							
	2015				2016			
	BA/total fungi		BR/total fungi		BA/total fungi		BR/total fungi	
	May	July	May	July	May	July	May	July
Control	29.43	8.90	5.24 × 10^−3^	8.50 × 10^−3^	0.00	0.00	3.73 × 10^−4^	7.74 × 10^−4^
*B. bassiana*	72.76	6.30	-	-	0.00	0.01	-	-
*B. brongniartii*	-	-	2.12 × 10^−2^	3.33 × 10^−2^	-	-	3.98 × 10^−4^	3.48 × 10^−3^
BA + BR	37.87	2.16	4.29 × 10^−4^	3.81 × 10^−3^	0.00	0.01	7.22	4.65 × 10^−1^

## Data Availability

The data presented in this study are available in the article and Supplementary Materials provided.

## Supplementary Materials

The following are available online at https://www.mdpi.com/2075-4450/12/2/127/s1. Table S1: Primers used for TRFLP analysis and qPCR assays, with annealing temperatures and target regions; Table S2: Results of the two-way ANOVA analysis of the data on damaged plants; Table S3: Data used in the principal component analysis to determine the relationship between plant damage, entomopathogenic fungi abundance, and climatic parameters at four time points in the two trials; Table S4: Average values of optical density (wavelength 490 nm) of *Beauveria bassiana* (BA), *Beauveria brongniartii* (BR), and of a co-inoculum (BABR) cultured on 95 carbon sources + water. The values are the average well color development of the whole Biolog FF plates (average of all substrates), after 72-h and 92-h incubation. One-way ANOVA, followed by Tukey’s test, was run to evaluate statistical significance of the differences (*p* < 0.001) that are marked with different letters; Table S5a: Average values of mitochondrial activity of *Beauveria bassiana* (BA), *Beauveria brongniartii* (BR), and of the co-inoculum (BABR) cultured on 95 carbon sources + water. The optical density values obtained at 490 nm wavelength of 4 biological replicates, after subtraction of the blank, were compared with One-way ANOVA, followed by Tukey’s test. Only the Biolog FF substrates that triggered a significantly different chitinases activity (see Table S6.1) are listed in this table; Table S5b: Mitochondrial activity (optical density values in FF Biolog plates after 96-h incubation, measured at 490 nm wavelength) of *Beauveria bassiana* (BA), *Beauveria brongniartii* (BR), and of the co-inoculum (BABR). The 96 substrates, including water, were classified into the fifteen functional groups listed in the table. The average absorbance for all wells in each group were calculated. Statistically significant differences (*p* < 0.01) between inocula are marked with different letters; Table S6a: NAGase activity of *Beauveria bassiana* (BA), *Beauveria brongniartii* (BR) and of the co-inoculum (BABR). The values are reported as relative fluorescence units (RFU). One-way ANOVA, followed by Tukey’s HSD test, was run on RFU subtracted by blank values. Only the Biolog FF substrates that triggered a statistically significant difference (within three biological replicates) between the treatments are listed. However, the average values of two substrates that gave no significant differences, but have a biological meaning (B11 D-glucosamine and A4 N-acetyl-D-glucosamine), are shown at the end of the table. Statistically significant differences (*p* < 0.01) in NAGase activity between inocula are marked with different letters; Table S6b: NAGase activity of *Beauveria bassiana* (BA), *Beauveria brongnartii* (BR), and of the co-inoculum (BABR). The 96 substrates were classified into the fifteen functional groups listed in the table. NAGase activity for all wells in each group were calculated. The values are reported as relative fluorescence units (RFU). The fluorescence values were compared with one-way ANOVA, followed by Tukey’s test. Statistically significant differences (*p* < 0.01) in NAGase activity between the inocula are marked with different letters; Table S7: Fungal genes copies number in the soil of the two trials (NW and BR). Data followed by the same letter are not significantly different at *p* ≤ 0.05 (Tukey’s HSD post-hoc test).
