# Supplementary material for: Biocontrol of Melolontha spp. Grubs in Organic Strawberry Plantations by Entomopathogenic Fungi as Affected by Environmental and Metabolic Factors and the Interaction with Soil Microbial Biodiversity
Source: Insects. 2021 Feb 2;12(2):127. doi: 10.3390/insects12020127 (PMC7912822; doi:10.3390/insects12020127)
Supplement: Supplementary file 1 [file insects-12-00127-s001.pdf]

Table S1. Primers used for TRFLP analysis and qPCR assays, with annealing temperatures and target regions.

Target Group	Primers	Primer Sequence	Amplicon Length (bp)	Annealing Temp (°C)	Reference	Target Regions
All Fungi (qPCR)	ITS1<br>5.8S	5′CTTGGTCATTTAGAGGAAGTAA3′<br>5′CGC TGC GTT CTT CAT CG3′	300 bp	53°C	Fierer et al. 2005	ITS1
All bacteria (TRFLP)	63F<br>1087R	5′AGGCCTAACACATGCAAGTC3′<br>5′CTCGTTGCGGGACTTACCCC3′	1000 bp		Singh et al., 2006	16S rRNA
All Fungi (TRFLP)	ITS1<br>ITS4	5′CTTGGTCATTTAGAGGAAGTAA3′<br>5′TCCTCCGCTTATTGATAT GC3′	700 bp	55°C	Gardens and Brums 1993	ITS1

Table S2. Results of the two-way ANOVA analysis of the data on damaged plants.

Source of Variation	% of Total Variation	SS	F[#]
	Trial NW		
Treatment	19.66	2102	60.5**
Season	68.80	6823	131.3**
Interaction	9.42	1008	6.5**
	Trial BZ		
Treatment	4.40	375	4.6*
Season	80.65	6878	104.0**
Interaction	6.19	528	2.7

[#] F value is significant for * $p \leq 0.05$ or ** $p \leq 0.01$.

Table S3. Data used in the principal component analysis to determine the relationship between plant damage, entomopathogenic fungi abundance and climatic parameters at four time points in the two trials.

Trial	Year	Season	Timepoint	Treatment	*B. bassiana* (CFU)	*B. brongniartii* (CFU)	Damaged Plants [%]	Average Temperature [°C]	Air Humidity [%]	Total Monthly Precipitation
NW	2014	Autumn	September	Control	0.33	0.00	21.3	14.2	79.9	29.2
				BA	1.33	0.00	8.4	14.2	79.9	29.2
				BR	1.00	3.00	11.7	14.2	79.9	29.2
				BA + BR	1.67	0.33	9.6	14.2	79.9	29.2
	2015	Spring	June	Control	1.67	0.00	5.2	16.7	70.7	18.0
				BA	4.67	0.00	2.0	16.7	70.7	18.0
				BR	0.67	0.67	1.5	16.7	70.7	18.0
				BA + BR	2.67	0.00	1.2	16.7	70.7	18.0
		Autumn	October	Control	0.33	0.00	29.5	6.8	81.7	46.8
				BA	1.67	0.67	13.7	6.8	81.7	46.8
				BR	0.67	6.00	13.7	6.8	81.7	46.8
				BA + BR	0.33	1.00	14.0	6.8	81.7	46.8
	2016	Spring	July	Control	2.00	0.00	46.5	18.9	73.3	138.2
				BA	1.00	0.00	36.5	18.9	73.3	138.2
				BR	0.33	0.00	22.5	18.9	73.3	138.2
				BA + BR	0.33	0.00	19.2	18.9	73.3	138.2
BZ	2015	Spring	June	Control	0.67	0.00	0.0	16.7	70.7	18.0
				BA	1.00	0.00	0.0	16.7	70.7	18.0
				BR	0.33	1.33	0.0	16.7	70.7	18.0
				BA + BR	0.67	1.00	0.0	16.7	70.7	18.0
		Autumn	October	Control	0.67	0.00	14.3	6.8	81.7	46.8
				BA	1.00	0.00	12.3	6.8	81.7	46.8
				BR	0.00	1.00	11.7	6.8	81.7	46.8
				BA + BR	1.67	4.00	12.0	6.8	81.7	46.8
	2016	Spring	July	Control	0.33	0.00	47.3	18.9	73.3	138.2
				BA	1.00	0.00	33.7	18.9	73.3	138.2
				BR	0.00	0.00	28.3	18.9	73.3	138.2
				BA + BR	0.67	1.33	24.7	18.9	73.3	138.2

Table S4. Average values of optical density (wavelength 490 nm) of *Beauveria bassiana* (BA), *Beauveria brongniartii* (BR) and of a co-inoculum (BABR) cultured on 95 carbon sources + water. The values are the average well colour development of the whole Biolog FF plates (average of all substrates), after 72-hour and 92-hour incubation. One-way ANOVA, followed by Tukey's test, was run to evaluate statistical significance of the differences ($p < 0.001$) that are marked with different letters.

	**72 h**	**96 h**
**BA**	0.38 a	0.76 a
**BABR**	0.38 a	0.75 a
**BR**	0.30 b	0.49 b
Pr > F (Model)	<0.0001	<0.0001
Significant	Yes	Yes

Table S5a Average values of mitochondrial activity of *Beauveria bassiana* (BA), *Beauveria brongniartii* (BR) and of the co-inoculum (BABR) cultured on 95 carbon sources + water. The optical density values obtained at 490 nm wavelength of 4 biological replicates, after subtraction of the blank, were compared with One-way ANOVA, followed by Tukey's test. Only the Biolog FF substrates that triggered a significantly different chitinases activity (see Table S6.1) are listed in this table.

	**BA**	**BABR**	**BR**	**Pr > F**	**Significant**
**A3 N-Acetyl-D-Galactosamine**	0.23 a	0.22 a	0.15 a	0.2513	No
**A2 Tween 80**	0.47 b	0.45 b	0.55 a	<0.0001	Yes
**A1 Water**	0.04 a	0.07 a	0.09 a	0.2513	No
**G5 Succinic Acid Mono-Methyl Ester**	0.31 a	0.14 ab	0.02 b	0.0398	Yes
**H9 Putrescine**	0.42 a	0.33 b	0.35 b	0.0291	Yes
**H8 2-Amino Ethanol**	0.21 a	0.12 ab	0.06 b	0.0933	Yes
**D10 D-Psicose**	0.17 a	0.08 b	0.16 a	0.0289	Yes
**F3 Fumaric Acid**	1.24 a	1.27 a	0.72 b	<0.0001	Yes
**F12 Quinic Acid**	0.07 a	0.07 a	0.06 a	0.1908	No
**E11 Xylitol**	0.22 ab	0.18 b	0.34 a	0.0263	Yes
**C7 2-Keto-D-Gluconic Acid**	0.55 b	0.59 b	0.76 a	0.0164	Yes
**C3 D-Glucuronic Acid**	0.11 a	0.08 a	0.09 a	0.6227	No
**B1 a- Cyclodextrin**	0.07 a	0.06 a	0.05 a	0.5552	No
**C8 a-D-Lactose**	0.17 a	0.06 b	0.12 ab	0.0105	Yes
**B2 b-Cyclodextrin**	0.02 a	0.02 a	0.01 a	0.3735	No
**F4 b-Hydroxy-butyric Acid**	0.09 a	0.05 a	0.10 a	0.1804	No
**B6 L-Fucose**	0.06 b	0.01 c	0.11 a	<0.0001	Yes
**A12 D-Cellobiose**	1.49 a	1.46 a	0.65 b	0.0006	Yes
**E5 L-Sorbose**	0.20 a	0.15 a	0.08 a	0.3880	No
**D5 a-Methyl-D-Galactoside**	0.05 a	0.05 a	0.07 a	0.5061	No
**B3 Dextrin**	0.97 a	0.72 b	0.24 c	<0.0001	Yes
**C9 Lactulose**	0.10 a	0.09 a	0.09 a	0.9783	No
**C10 Maltilol**	0.08 ab	0.04 b	0.09 a	0.0477	Yes
**G6 N-Acetyly-L-Glutamic Acid**	0.11 c	0.17 b	0.30 a	0.0002	Yes
**D8 b-Methyl-D-Glucoside**	0.99 a	0.80 b	0.51 c	0.0005	Yes
**B4 i-Erythritol**	0.56 a	0.26 b	0.28 b	0.0011	Yes
**D12 L-Rhamnose**	0.07 b	0.10 a	0.10 a	0.0002	Yes
**C4 Glycerol**	1.47 a	1.20 b	0.86 c	<0.0001	Yes
**E3 Sedoheptulosan**	0.05 a	0.07 a	0.07 a	0.6758	No
**G1 D-Saccharin Acid**	0.05 a	0.06 a	0.06 a	0.9051	No
**A8 D-Arabinose**	0.09 a	0.10 a	0.08 a	0.5930	No
**F9 L-Lactic Acid**	0.10 a	0.02 b	0.09 a	0.0001	Yes
**D4 D-Melibiose**	0.23 a	0.24 a	0.06 b	0.0006	Yes
**A6 Adonitol**	1.16 a	1.15 a	0.41 b	<0.0001	Yes
**B7 D-Galactose**	0.92 a	0.88 a	0.40 b	<0.0001	Yes
**G11 L-Aspartic Acid**	1.07 b	1.19 a	0.72 c	<0.0001	Yes
**E8 D-Tagatose**	0.05 ab	0.04 b	0.09 a	0.0331	Yes
**F8 D-Lactic Acid Methyl Ester**	0.07 a	0.02 b	0.10 a	0.0004	Yes

**D3 D-Melezitose**	0.86 a	0.67 b	0.13 c	<0.0001	Yes
**E1 D-Ribose**	0.34 a	0.32 b	0.23 c	<0.0001	Yes
**F1 g-Amino-butyric Acid**	1.19 a	0.86 b	0.12 c	<0.0001	Yes
**B9 Gentibiose**	1.35 a	1.25 b	0.71 c	<0.0001	Yes
**D1 D-Mannitol**	1.05 b	1.15 a	0.60 c	<0.0001	Yes
**G10 L-Asparagine**	1.24 a	1.21 a	0.27 b	<0.0001	Yes
**E9 D-Trehalose**	1.56 a	1.33 b	0.74 c	<0.0001	Yes
**E12 D-Xylose**	0.25 b	0.23 c	0.27 a	0.0002	Yes
**E4 D-Sorbitol**	0.91 a	0.76 a	0.35 b	<0.0001	Yes
**A7 Amygdalin**	0.67 a	0.61 b	0.38 c	<0.0001	Yes
**C6 m-Inositol**	0.55 a	0.48 b	0.21 c	<0.0001	Yes
**E10 Turanose**	1.47 a	1.32 b	0.27 c	<0.0001	Yes
**A10 D-Arabitol**	0.37 a	0.34 a	0.34 a	0.5874	No
**G8 L-Alanine**	1.22 b	1.33 a	0.95 c	<0.0001	Yes
**G9 L-Alanyl-Glycine**	1.30 a	1.35 a	1.12 b	0.0059	Yes
**H4 L-Proline**	0.90 a	0.89 a	0.31 b	<0.0001	Yes
**C2 Glucuronamide**	0.03 a	0.02 a	0.04 a	0.3711	No
**B8 D-Galacturonic Acid**	0.05 a	0.02 b	0.00 b	0.0005	Yes
**F7 a-Keto-glutaric Acid**	0.83 a	0.79 a	0.62 b	0.0126	Yes
**B11 D-Glucosamine**	0.34 a	0.24 a	0.25 a	0.0943	No
**A4 N-Acetyl-D-Glucosamine**	2.02 a	1.90 a	0.73 b	<0.0001	Yes

Table S5b Mitochondrial activity (optical density values in FF Biolog plates after 96-hour incubation, measured at 490 nm wavelength) of *Beauveria bassiana* (BA), *Beauveria brongniartii* (BR) and of the co-inoculum (BABR). The 96 substrates, including water, were classified into the fifteen functional groups listed in the table. The average absorbance for all wells in each group were calculated. Statistically significant differences ($p < 0.01$) between inocula are marked with different letters.

	BA	BABR	BR	*p*	Significant
**Water**	0.04 a	0.07 a	0.09 a	0.2513	No
**Heptoses**	0.05 a	0.07 a	0.07 a	0.6758	No
**Hexoses**	0.64 a	0.58 b	0.31 c	<0.0001	Yes
**Pentoses**	0.22 a	0.20 ab	0.17 b	0.0387	Yes
**Sugar acids**	0.17 a	0.15 a	0.19 a	0.1633	No
**Hexosamines**	0.66 a	0.61 b	0.29 c	<0.0001	Yes
**Polyols**	0.79 a	0.69 b	0.42 c	<0.0001	Yes
**Polysaccharides**	0.32 a	0.24 a	0.12 b	0.0015	Yes
**Oligosaccharides**	0.77 a	0.67 a	0.25 b	<0.0001	Yes
**Glucosides**	0.76 a	0.63 b	0.35 c	<0.0001	Yes
**Peptides**	1.27 b	1.36 a	1.05 c	<0.0001	Yes
**L-amino acids**	0.90 a	0.93 a	0.44 b	<0.0001	Yes
**Biogenic and heterocyclic amines**	0.20 a	0.14 a	0.15 a	0.2170	No
**TCA-cycle intermediates**	0.99 a	0.89 b	0.51 c	<0.0001	Yes
**Aliphatic organic acids**	0.15 a	0.07 b	0.14 ab	0.0687	Yes
**Other compounds**	0.19 a	0.15 b	0.16 b	0.0020	Yes

Table S6a NAGase activity of *Beauveria bassiana* (BA), *Beauveria brongniartii* (BR) and of the co-inoculum (BABR). The values are reported as Relative Fluorescence Units (RFU). One-way ANOVA, followed by Tukey's HSD test, was run on RFU subtracted by blank values. Only the Biolog FF substrates that triggered a statistically significant difference (within three biological replicates) between the treatments are listed. However, the average values of two substrates that gave no significant differences, but have a biological meaning (B11 D-Glucosamine and A4 N-Acetyl-D-Glucosamine), are shown at the end of the table. Statistically significant differences (p <0.01) in NAGase activity between inocula are marked with different letters.

	BABR	BA	BR	*p*	Significant
**A3 N-Acetyl-D-Galactosamine**	38973.83 a	30164.10 b	7657.23 c	<0.0001	Yes
**A2 Tween 80**	8099.08 a	318.03 b	2201.43 b	0.00024	Yes
**A1 Water**	5285.63 a	7240.15 a	1237.63 b	0.00169	Yes
**G5 Succinic Acid Mono-Methyl Ester**	4938.63 a	989.94 b	748.55 b	<0.0001	Yes
**H9 Putrescine**	4517.13 a	3644.76 a	1550.39 b	0.01127	Yes
**H8 2-Amino Ethanol**	3857.01 a	2850.70 b	2342.60 b	0.00911	Yes
**D10 D-Psicose**	3652.97 a	2494.84 b	1212.21 c	<0.0001	Yes
**F3 Fumaric Acid**	3560.05 a	2381.12 b	1202.73 c	0.00699	Yes
**F12 Quinic Acid**	3513.47 a	3724.78 a	992.91 b	0.02497	Yes
**E11 Xylitol**	3476.47 a	2150.39 b	1488.98 c	<0.0001	Yes
**C7 2-Keto-D-Gluconic Acid**	3106.24 a	3081.86 a	1234.45 b	0.00082	Yes
**C3 D-Glucuronic Acid**	3039.83 a	1377.94 b	1083.87 b	<0.0001	Yes
**B1 a- Cyclodextrin**	2968.93 b	3653.38 a	865.37 c	<0.0001	Yes
**C8 a-D-Lactose**	2836.76 a	1148.71 b	1459.78 b	0.00881	Yes
**B2 b-Cyclodextrin**	2697.16 a	309.73 b	739.28 b	0.0001	Yes
**F5 g-Hydroxy-butyric Acid**	2674.79 a	1283.67 b	1314.19 b	0.00579	Yes
**B6 L-Fucose**	2599.36 a	1258.59 b	1346.91 b	<0.0001	Yes
**A12 D-Cellobiose**	2594.99 b	2823.62 b	6585.87 a	0.02484	Yes
**E5 L-Sorbose**	2594.17 a	1103.22 a	1352.57 a	0.04775	Yes
**D5 a-Methyl-D-Galactoside**	2339.04 a	1716.34 ab	1123.98 b	0.01646	Yes
**B3 Dextrin**	2335.29 b	1327.88 c	3281.67 a	0.00016	Yes
**C9 Lactulose**	2301.62 a	1224.54 b	889.98 b	0.00018	Yes
**C10 Maltilol**	2281.81 a	1445.29 b	556.54 c	0.00069	Yes
**G6 N-Acetyly-L-Glutamic Acid**	2207.02 b	3232.20 a	2551.94 b	0.01043	Yes
**D8 b-Methyl-D-Glucoside**	2163.32 a	2507.93 a	905.93 b	0.00021	Yes
**B4 i-Erythritol**	2102.57 a	949.29 b	2133.36 a	<0.0001	Yes
**D12 L-Rhamnose**	2052.14 a	2061.98 a	728.12 b	0.01566	Yes
**C4 Glycerol**	2024.52 a	2388.27 a	1609.63 b	0.00894	Yes
**E3 Sedoheptulosan**	1974.46 a	862.99 b	684.57 c	<0.0001	Yes
**G1 D-Saccharin Acid**	1905.42 a	915.64 b	1202.77 b	0.00398	Yes
**A8 D-Arabinose**	1887.48 a	1689.88 ab	1386.32 b	0.03753	Yes
**F9 L-Lactic Acid**	1832.58 a	1056.54 b	892.29 b	0.01149	Yes
**D4 D-Melibiose**	1766.54 ab	2979.96 a	882.67 b	0.03593	Yes
**A6 Adonitol**	1654.44 b	592.59 c	2496.35 a	0.00132	Yes
**B7 D-Galactose**	1587.43 a	1543.29 a	733.28 b	0.01243	Yes
**G11 L-Aspartic Acid**	1486.39 b	1056.79 b	2397.61 a	0.00551	Yes
**E8 D-Tagatose**	1319.66 a	667.20 b	868.74 b	0.00144	Yes
**F8 D-Lactic Acid Methyl Ester**	1293.46 a	839.14 b	875.95 b	0.02778	Yes
**D3 D-Melezitose**	1252.92 a	593.54 b	1291.60 a	0.00248	Yes
**E1 D-Ribose**	1209.79 b	1262.34 b	1755.11 a	0.02063	Yes
**F1 g-Amino-butyric Acid**	1201.95 b	3219.10 a	841.19 b	<0.0001	Yes
**B9 Gentibiose**	1170.61 c	2102.09 b	3703.12 a	<0.0001	Yes
**D1 D-Mannitol**	1169.76 b	5238.52 a	2063.18 b	0.00025	Yes
**G10 L-Asparagine**	1155.01 b	898.67 b	1866.82 a	0.00068	Yes
**E9 D-Trehalose**	1133.19 b	2019.31 a	1795.83 a	0.00343	Yes
**E12 D-Xylose**	1103.67 b	749.73 b	2977.18 a	<0.0001	Yes
**E4 D-Sorbitol**	1092.78 c	1332.66 b	2909.99 a	<0.0001	Yes
**A7 Amygdalin**	995.82 b	657.03 b	5083.26 a	<0.0001	Yes
**C6 m-Inositol**	964.53 a	436.34 b	309.46 b	0.0023	Yes
**E10 Turanose**	898.92 b	2377.42 a	1132.85 b	0.02927	Yes
**A10 D-Arabitol**	752.93 b	849.79 b	3938.66 a	<0.0001	Yes
**G8 L-Alanine**	711.28 b	801.37 b	2100.08 a	0.00013	Yes
**G9 L-Alanyl-Glycine**	711.28 b	801.37 b	2100.08 a	0.00013	Yes
**H4 L-Proline**	655.44 b	741.14 b	3283.95 a	<0.0001	Yes

**C2 Glucuronamide**	621.47 a	313.36 b	495.84 ab	0.02185	Yes
**B8 D-Galacturonic Acid**	486.25 b	343.17 c	568.45 a	0.00025	Yes
**F7 a-Keto-glutaric Acid**	313.35 c	6324.80 a	2033.20 b	<0.0001	Yes
**B11 D-Glucosamine**	17913.97 a	10810.75 a	6008.89 a	0.12	No
**A4 N-Acetyl-D-Glucosamine**	6665.80 a	6839.85 a	4835.83 a	0.85	No

Table S6b NAGase activity of *Beauveria bassiana* (BA), *Beauveria brongnartii* (BR) and of the co-inoculum (BABR). The 96 substrates were classified into the fifteen functional groups listed in the table. NAGase activity for all wells in each group were calculated. The values are reported as Relative Fluorescence Units (RFU). The fluorescence values were compared with One-way ANOVA, followed by Tukey's test. Statistically significant differences ($p < 0.01$) in NAGase activity between the inocula are marked with different letters.

	**BA**	**BABR**	**BR**	***p***	**Significant**
**Water**	7240.15 a	5285.63 a	1237.63 b	0.0017	Yes
**Heptoses**	862.99 b	1974.46 a	684.57 c	<0.0001	Yes
**Hexoses**	1722.84 a	1798.74 a	1159.99 a	0.1613	No
**Pentoses**	1584.60 b	1926.65 a	1815.73 a	0.0147	Yes
**Sugar acids**	1222.34 b	1742.16 a	1066.84 b	0.0048	Yes
**Hexosamines**	12164.24 b	16312.28 a	5040.47 c	0.0008	Yes
**Polyols**	1742.23 b	1654.75 b	2118.70 a	0.0053	Yes
**Polysaccharides**	1885.11 b	2873.27 a	1665.21 b	0.0003	Yes
**Oligosaccharides**	2013.68 a	2231.57 a	1862.79 a	0.5638	No
**Glucosides**	1353.80 a	1302.81 a	1661.80 a	0.1130	No
**Peptides**	1477.16 b	1691.30 b	2164.24 a	0.0082	Yes
**L-amino acids**	1553.05 b	1542.81 b	2079.00 a	0.0047	Yes
**Biogenic and heterocyclic amines**	2433.37 a	2893.09 a	1461.13 b	0.0059	Yes
**TCA-cycle intermediates**	2751.69 a	2249.88 a	1589.54 b	0.0037	Yes
**Aliphatic organic acids**	987.14 c	1642.57 a	1133.71 b	<0.0001	Yes
**Other compounds**	1354.59 b	2781.51 a	1567.34 b	0.0012	Yes

Table S7. Fungal genes copies number in the soil of the two trials (NW and BR). Data followed by the same letter are not significantly different at $p \leq 0.05$ (Tukey's HSD post-hoc test).

**Treatment**	**Trial NW**		**Trial BZ**	
	**October 2015**	**July 2016**	**October 2015**	**July 2016**
Control	2907421 a	6182248 a	1351806 ab	5516163 a
B, bassiana	973790 b	5688618 a	1829224 a	3051788 b
B, brongniartii	2366551 ab	5939836 a	984140 ab	1501112 c
BA + BR	1300206 ab	5936636 a	875966 b	5934904 a
